# Cost and effectiveness of one session treatment (OST) for children and young people with specific phobias compared to multi-session cognitive behavioural therapy (CBT): results from a randomised controlled trial

**DOI:** 10.1186/s12888-022-04192-8

**Published:** 2022-08-12

**Authors:** Han-I. Wang, Barry Wright, Lucy Tindall, Cindy Cooper, Katie Biggs, Ellen Lee, M. Dawn Teare, Lina Gega, Alexander J. Scott, Emily Hayward, Kiera Solaiman, Thompson Davis, Dean McMillan, Simon Gilbody, Steve Parrott

**Affiliations:** 1 Department of Health Sciences, Seebohm Rowntree Building, University of York, Heslington, YO10 5DD York UK; 2 Leeds and York Partnership NHS Foundation Trust, Leeds, UK; 3 University of Sheffield, Sheffield, UK; 4 Newcastle University, Newcastle upon Tyne, UK; 5 Hull York Medical School, York, UK; 6 Keele University, Keele, UK; 7 Louisiana State University, Baton Rouge, USA; 8 The University of Alabama, Tuscaloosa, USA

**Keywords:** Cost-effectiveness, Child, Adolescent, Phobic disorders, Mental health services, Cost, EQ-5D-Y, One session treatment, Cognitive behavioural therapy

## Abstract

**Background:**

In the UK, around 93,000 (0.8%) children and young people (CYP) are experiencing specific phobias that have a substantial impact on daily life. The current gold-standard treatment—multi-session cognitive behavioural therapy (CBT) – is effective at reducing specific phobia severity; however, CBT is time consuming, requires specialist CBT therapists, and is often at great cost and limited availability. A briefer variant of CBT called one session treatment (OST) has been found to offer similar clinical effectiveness for specific phobia as multi-session CBT. The aim of this study was to assess the cost-effectiveness of OST compared to multi-session CBT for CYP with specific phobias through the Alleviating Specific Phobias Experienced by Children Trial (ASPECT), a two-arm, pragmatic, multi-centre, non-inferiority randomised controlled trial.

**Methods:**

CYP aged seven to 16 years with specific phobias were recruited nationally via Health and Social Care pathways, remotely randomised to the intervention group (OST) or the control group (CBT-based therapies) and analysed (*n* = 267). Resource use based on NHS and personal social services perspective and quality adjusted life years (QALYs) measured by EQ-5D-Y were collected at baseline and at six-month follow-up. Incremental cost-effectiveness ratio (ICER) was calculated, and non-parametric bootstrapping was conducted to capture the uncertainty around the ICER estimates. The results were presented on a cost-effectiveness acceptability curve (CEAC). A set of sensitivity analyses (including taking a societal perspective) were conducted to assess the robustness of the primary findings.

**Results:**

After adjustment and bootstrapping, on average CYP in the OST group incurred less costs (incremental cost was -£302.96 (95% CI -£598.86 to -£28.61)) and maintained similar improvement in QALYs (QALYs gained 0.002 (95% CI − 0.004 to 0.008)). The CEAC shows that the probability of OST being cost-effective was over 95% across all the WTP thresholds. Results of a set of sensitivity analyses were consistent with the primary outcomes.

**Conclusion:**

Compared to CBT, OST produced a reduction in costs and maintained similar improvement in QALYs. Results from both primary and sensitivity analyses suggested that OST was highly likely to be cost saving.

**Trial registration:**

ISRCTN19883421 (30/11/2016).

**Supplementary Information:**

The online version contains supplementary material available at 10.1186/s12888-022-04192-8.

## Introduction

A specific phobia is a type of anxiety disorder defined as an intense, persistent and uncontrollable fear of an identifiable object, situation or activity (e.g. dogs, heights or injections) that leads to a high degree of anxiety, distress, and avoidance [1]. It is one of the most common mental health difficulties in the UK, with an estimated 93,000 (0.8%) children and young people (CYP) experiencing specific phobias, severe enough to affect their daily activity [2, 3].

Multi-session cognitive behavioural therapy (CBT) is the current gold-standard treatment for specific phobias [4–6], typically delivered over six to 12 one-hour sessions. It is effective and has a robust evidence-base [7–9]. However, CBT is time consuming, costly and has limited availability. One session treatment (OST), on the other hand, has been found to have potential to offer a brief and clinically effective treatment for specific phobias [10]. This is because OST, a variant of CBT [11], shares many of the same principles as CBT but does not require an extensive treatment period [12, 13]. Instead, OST is delivered over two sessions: an assessment and planning session lasting approximately one hour, and a second exposure session typically lasting up to three hours.

Few large-scale randomised controlled trials (RCTs) have investigated the clinical effects of OST for CYP with specific phobias [14–16]. Despite the differences in sample size, all three studies suggested that OST was superior to control groups (including wait-list and educational support) and provided significant clinical improvements after treatment. The recent Alleviating Specific Phobias Experienced by Children Trial (ASPECT) [17] tested the efficacy of OST compared to routinely delivered multi-session CBT and found that OST and multi-session CBT offer similar clinical benefit [18]. However, to our knowledge, no studies have investigated the cost-effectiveness of OST for CYP with specific phobias.

To bridge the evidence gap, the aim of the current study was to assess the cost-effectiveness of OST compared to CBT in CYP with specific phobias using a within-trial cost-utility analysis from the UK NHS and Personal Social Services (PSS) perspective. To take the full economic impact into account, an additional economic evaluation from the societal perspective was also included in the sensitivity analysis. This paper reports the economic evaluation results of OST for CYP with specific phobias conducted as part of the ASPECT [17].

## Methods

### Trial design and participants

This economic evaluation was embedded in ASPECT, a two-arm, pragmatic, multi-centre, non-inferiority RCT comparing OST with CBT for CYP with specific phobia. Details of the ASPECT methods have been published elsewhere [17]. In summary, CYP aged seven to 16 years with specific phobia were recruited nationally via Health and Social Care pathways (i.e. Child and Adolescent Mental Health Services (CAMHS), voluntary agencies, school-based wellbeing services and a University-based CYP wellbeing service) between June 2017 and January 2020. The presence of a specific phobia was assessed by DSM-5 criteria [1] using the Anxiety Disorder Interview Schedule (ADIS) [19, 20]. CYP were excluded if exposing the person to the phobic stimulus would be unsafe, or where a phobia was deemed by a clinician to be unsuitable for exposure therapy. CYP with co-morbidities (e.g. autism spectrum disorders) were included. Informed consent and baseline measurements were obtained and completed prior to randomisation. These included a face-to-face measure: the Behavioural Avoidance Task (BAT), ADIS, Child Anxiety Impact Scale (CAIS), and Revised Children’s Anxiety and Depression Scale (RCADS), and a health related quality of life (HRQoL) outcome measure: the youth version of the EQ-5D (EQ-5D-Y) and the Child Health Utility-9D (CHU-9D). Following completion of baseline measures, eligible CYP were remotely randomised to either the intervention group (OST) or the control group receiving usual care (CBT-based therapies) (1:1) using an online system through the Trials Unit. All CYP were followed up six-months after randomisation where all outcome measures were repeated. A flowchart of the study can be found in Additional file 1: Appendix 1. In total, 268 CYP (134 per arm) were recruited and randomised. This exceeded the revised sample size target, and is sufficient to detect a standardised non-inferiority margin of 0.4 on the primary outcome measure [21] with a power in excess of 90% based on intraclass correlation coefficient of 0.7, an interim observed dropout rate of 27.3%, the finding that each therapist was treating five CYP and with a design effect of 1.04.

### Interventions

CBT uses cognitive and behavioural techniques to support individuals to change unhelpful behaviours and thought patterns arising in feared situations [4, 8, 22, 23]. CBT-based interventions are typically delivered in weekly hour-long sessions comprising the usual practices of building a fear hierarchy, exposure and cognitive restructuring. Each CBT session has a specific agenda and sets homework tasks for the CYP between sessions. There is no recommended number of CBT sessions for specific phobias, however, CYP would usually receive six to 12 sessions.

OST is a variant of CBT but takes a more condensed and intensive approach. OST typically involves a combination of treatment techniques, focusing on graded exposure supplemented by participant modelling, reinforcement, exploration of cognitions in the context of behavioural experiments, and skills training [24]. Unlike CBT, OST comprises two sessions: 1) an initial functional assessment and co-planning session lasting around one hour; and 2) a session (typically lasting around three hours) involving graded exposure to the phobic stimulus until fear subsides, with or without active exploration of fear-related thoughts. The main treatment session is structured around a series of graded exposure tasks, starting from the least threatening situation and increasing in difficulty as the session goes along [12], and has sets homework tasks after the session. OST has been shown to be clinically efficacious in CYP [13, 15, 16, 25].

### Outcome measurements

The health economic outcome measurements for this study were quality adjusted life years (QALYs) measured by the EQ-5D-Y (self-complete version) [26] and the CHU-9D [27]. The EQ-5D-Y is a five-item questionnaire for self-completion by CYP aged eight to 15 years. It measures HRQoL on five dimensions (mobility, self-care, doing usual activities, having any pain or discomfort, and feeling worried) with three response levels (no problems, some problems, and extreme problems). The measure has been shown to be a reliable and valid instrument for use in CYP [26]. In this study, 11 CYP were aged seven and were, thus, under the age limit of the self-complete version (eight to 15 years old). These CYP were asked to complete the EQ-5D-Y questionnaire as if they belonged to the CYP group aged eight to 15, for consistency reasons. The CHU-9D, a child-completed, nine-item questionnaire also measures HRQoL for CYP aged seven to 17 years. Participants describe their feelings on nine dimensions (worried, sad, pain, tired, annoyed, schoolwork/homework, sleep, daily routine, and able to join in activities) by selecting one of five response levels (no problems, a few problems, some problems, many problems, and extreme problems) [27]. Both instruments provide utility values that allow the calculation of QALYs for use in cost-utility analysis. However, QALYs measured by EQ-5D-Y using UK adult population tariffs was chosen for primary analysis, as EQ-5D is the preferred instrument for the National Institute for Health and Care Excellence (NICE) [28].

To measure QALYs, individual responses to the EQ-5D-Y and CHU-9D were first converted to utilities based on UK adult population valuation sets [29, 30], which was chosen due to lack of UK CYP population valuation sets. Then, the estimated utilities at baseline and six-month follow-up point were further joined to calculate QALYs using the area under the curve (AUC) approach [31]. The AUC method assumes that there is a linear relationship between utilities at different time points. Hence, to calculate QALYs, the two utility scores for each individual were first averaged and then multiplied by the duration between the two scores (six months). The non-health economic related outcome measurements of the ASPECT trial are described elsewhere [18].

### Cost measurements

Both the NHS and personal social service (NHS/PSS) perspective and the societal perspective were considered in this study. Costs from the NHS/PSS perspective included costs related to healthcare and social services, while societal perspective additionally considered costs of education-related services, parental out-of pocket expenses (i.e. private treatments), and parental productivity costs (time off work due to care for CYP’s phobia condition).

### Resource use measurement

All resource use incurred during the six-month follow-up was considered in this study, including both intervention and service use required by CYP with specific phobias. Resource use information for training and intervention delivery was collected using tailored questionnaires completed by the study team and therapists, respectively. The resource use required to train professionals in OST was measured by the time spent by the trainer and included travel costs and the cost of materials used for the training. Costs associated with delivering the intervention were also measured by the time spent by professionals as well as other resources used (including second therapist, administration, preparation, supervision and phobic stimulus acquisition, e.g. animal hire). Information related to overheads and facility was not collected, as these costs have been allocated to the staff time and reflected in the unit costs [32].

Service use data were collected using tailored resource utilisation questionnaires completed by parents/guardians. The questionnaires were specifically designed for ASPECT and based upon previous studies focusing on CYP with mental health issues [33–35]. Compared to previous resource use questionnaires, more open questions were added. This was in order to collect detailed information about the resource use outside the healthcare and education systems, such as the privately paid mental health services. Overall, service use included parent-reported use of primary and secondary healthcare, as well as social care. Medication usage was also included by collecting information like name and dosage of the medicine, start and end dates, and the administration frequency. Additional therapies and services received in either arm during the six-month follow-up period were recorded, and the duration was assumed to be one hour based on expert opinions. Data on productivity loss due to work absenteeism to care for the CYP were also collected.

### Valuation of resource use

All the resource use data were further multiplied by corresponding unit cost to arrive at total costs in each arm using the bottom-up costing approach. Unit costs of health and social service use were obtained from the UK national database of National Cost Collection 2018/19 (previously called Reference Costs) [28] and the Unit Costs of Health and Social Care 2018 produced by the Personal Social Services Research Unit (PSSRU) [32]. Unit costs of medication were based on the Prescription Cost Analysis – England 2018 [36]. Privately paid mental health services were separately estimated via market prices based on the information from the national online psychiatry service [37], while parental productivity costs were valued according to national average wage rates [38].

All costs were expressed in 2018 UK sterling. Discounting of costs and QALYs was not applied, as the study timeframe was less than one year [39].

### Missing data

All eligible CYP who had both utility and cost data at baseline and six-month follow-up point are referred to as complete case. The complete cases along with the eligible CYP who had missing utility or cost data but had completed baseline assessments are referred to as base case. The identified missing utility and cost data were imputed using multiple imputation method via chained Eqs. [40]. The imputation were based on the following variables: trial arm, age, gender, study site, phobia type, underlying mental health conditions (autism spectrum disorder and attention deficit hyperactivity disorder), EQ-5D-Y utility scores, cost and ADIS Clinician Severity Rating (CRS) scores at baseline. These variables were available at baseline and were included in order to avoid missing any key information [41].

### Statistical and economic analyses

The primary analysis of this study was a within-trial cost-utility analysis that calculated incremental cost-effectiveness ratio (ICER) based on the costs from the NHS/PSS perspective and the QALYs measured by EQ-5D-Y. Costs and utilities for each CYP were measured at baseline and 6-month follow-up point. Hence, the study time horizon for this within-trial cost-utility analysis was six months.

To account for uncertainty, seemingly unrelated regression equations (SURE) that controlled for baseline utility [42], cost, age, gender, study site, phobia type and ADIS CSR score at baseline were bootstrapped 5,000 times. The SURE approach considers the distribution of the dependent variable as well as the correlation between cost and QALY outcomes [31]; while non-parametric bootstrap re-sampling method was suggested by Briggs and colleagues [43], as the distribution of regression residuals was likely to be skewed [44]. The number of 5000 iterations was chosen because it was considered to be sufficient to generate robust estimates of standard errors [43] and is widely used in trial-based cost-effectiveness analyses for mental health illness [45–47]. Covariates, such as baseline utility, cost, age, gender, and study site, were chosen based on the related cost utility analysis (CUA) study for mental health illness [47]. Phobia type and ADIS CSR scores were chosen because they reflect the disease type and severity, which are considered by experts to be relevant to costs and QALYs.

The 5,000 bootstrapped results were presented graphically on the cost-effectiveness plane (CE-plane), and the probability of OST being cost-effective against a range of willingness-to-pay (WTP) thresholds was depicted using a cost-effectiveness acceptability curve (CEAC) [48]. A range of possible WTP thresholds has been proposed to assess whether an intervention is worthwhile [49–51]. In this study, the national WTP threshold of £20,000-£30,000 per QALY gained suggested by NICE was used to decide whether OST is cost-effective compared to CBT [39].

A set of sensitivity analyses were conducted to test assumptions made in the primary analysis and to assess the robustness of our primary findings. First, a CUA using the complete case was conducted to assess the impact of the missing data. Second, a CUA was performed on those who received interventions within the follow-up period to assess the impact of the COVID-19 pandemic. This was done because some randomised CYP did not manage to receive any intervention sessions during the study period due to the COVID-19 pandemic (*n* = 67). Third, a CUA was performed from a societal perspective to account for all the economic impact outside the NHS/PSS perspective. Finally, a CUA that used the CHU-9D to estimate QALYs based on the UK population tariff [17] was conducted to assess the impact of outcome measurement instrument.

All analyses were pre-defined in the health economics analysis plan and were performed using Stata version 16 (StataCorp, College Station, Texas, USA).

### Ethical approval and informed consent

This study was funded by the National Institute for Health Research (NIHR) Health Technology Assessment programme (HTA15/38/04), and the International Standard Randomised Controlled Trial Number is ISRCTN19883421 (30/11/2016) [52]. The ethical approval was obtained from North East – York Ethics Research Committee (17/NE/0012), and the written informed consent was obtained from parents/guardians, alongside consent or assent from their child.

## Results

### Participants

A total of 340 CYP with specific phobias were recruited. After removing 72 ineligible CYP (Additional file 1: Appendix 1) and one CYP who was not eligible for multiple imputation due to missing baseline utilities, 267 CYP with specific phobia were randomised and available for primary analysis (133 were allocated to OST and 134 to CBT). This sample constitutes the base-case group. Among them, 190 (71.2%) CYP had both EQ-5D-Y and resource use data (from the NHS and PSS perspective) at both data collection time points. This sample constitutes the complete-case group. The details about the missing data and patterns on resource use and utility are reported in Additional file 1: Appendix 2.

The baseline characteristics of both base case and complete case can be found in Table 1. Slightly over one third of the CYP in the OST and the CBT arms were male, and more than 50% of CYP in both arms were of secondary school age (ranging from 11 to 16 years old). Differences in the ADIS CSR scores and EQ-5D-Y utility scores at baseline were small. Overall, the baseline characteristics were balanced across arms and samples.

**Table 1:** Baseline characteristics by trial arm

Baseline characteristics	Base case (*n* = 267)		Complete case (*n* = 190)	
	**OST (*N* = 133)**	**CBT (*N* = 134)**	**OST (*N* = 94)**	**CBT (*N* = 96)**
Gender, n (%)				
Male	47 (35.3%)	53 (39.6%)	34 (36.2%)	40 (41.7%)
Age (years), n (%)				
7–11	45 (33.8%)	39 (29.1%)	35 (37.2%)	30 (31.3%)
11–16	88 (66.2%)	95 (70.9%)	59 (62.8%)	66 (68.7%)
Mean (sd)	12.0 (2.6)	11.9 (2.6)	11.7 (2.6)	11.7 (2.6)
Ethnicity				
British	126 (94.7%)	129 (96.3%)	89 (94.7%)	94 (98.0%)
Non-British	7 (5.3%)	4 (3.0%)	5 (5.3%)	1 (1.0%)
Prefer not to say	-	1 (0.7%)	-	1 (1.0%)
Phobia type				
Animals	38 (28.5%)	41 (30.6%)	29 (30.9%)	34 (35.4%)
Blood-injection or injury	36 (27.1%)	32 (23.9%)	21 (22.3%)	23 (24.0%)
Vomit	36 (27.1%)	41 (30.6%)	22 (23.4%)	25 (26.0%)
Other	23 (17.3%)	20 (14.9%)	22 (23.4%)	14 (14.6%)
ADIS CSR				
Mean (sd)	7.6 (0.9)	7.5 (0.9)	7.6 (0.9)	7.5 (0.9)
EQ-5D-Y				
Mean (sd)	0.84 (0.12)	0.85 (0.12)	0.85 (0.11)	0.85 (0.13)
Site, n (%)				
North west England	70 (52.6%)	71 (53.0%)	58 (61.7%)	56 (58.3%)
East of England	35 (26.3%)	21 (15.7%)	17 (18.0%)	16 (16.7%)
Yorkshire and Humber	18 (13.5%)	30 (22.4%)	13 (13.8%)	18 (18.7%)
South west England	7 (5.3%)	6 (4.5%)	4 (4.3%)	4 (4.2%)
West Midlands	2 (1.5%)	5 (3.7%)	1 (1.1%)	2 (2.1%)
North West England	1 (0.8%)	1 (0.7%)	1 (1.1%)	-
Baseline costs (in 6-month period prior to randomisation, NHS/PSS perspective)				
Mean (sd)	596.48 (1039.42)	524.12 (1142.00)	590.80 (743.88)	567.44 (1311.69)

^Note:^ ADIS: anxiety disorder interview schedule; CRS: clinician severity rating; NHS: national health service; PSS: personal social services

### Costs

On average, the estimated intervention costs per session per CYP for OST and CBT were £184.26 (£62.19 for training and £122.07 for intervention delivery) and £58.59 (£0 for training and £58.59 for intervention delivery), respectively. The training cost of OST contained the costs for both “train the trainers” and “therapist training” sessions. The main cost driver of training costs was trainer-related fees, which accounted for near 90% of total training expenditure of OST. On the other hand, the training cost for CBT was zero, as therapists in both arms are already trained for CBT. In terms of the intervention delivery costs, it contained costs for session preparation, administration, delivery and additional resources (such as second therapists and stimuli). The main drivers of intervention delivery costs were those associated with the therapist time for delivering the intervention, which account for 68% and 79% for the total delivery cost of OST and CBT, respectively (Additional file 1: Appendix 3). On average, the total intervention cost (training and intervention delivery costs) is lower for OST (£209.60, sd: 150.43) than CBT (£287.71, sd: 263.63), as OST requires less sessions than multi-session CBT.

In terms of service costs, a summary of resource use over six months is shown in Additional file 1: Appendix 4, and the total costs broken down by perspective, type of service, trial arm and before and after imputation are presented in Table 2. As shown, the average costs for healthcare services and education services were similar between the two arms. CYP in the OST arm also incurred slightly lower average costs in private expenses and parental productivity losses compared to those in the CBT arm. Overall, CYP in the OST arm incurred lower average costs in both NHS/PSS and societal perspectives. This is observed in both the complete case and the base case. However, it is worth noting that some cost differences were likely to have been driven by the high cost cases. For instance, the slightly higher average cost of CAMH services in the CBT arm was driven by one high cost case (25 psychotherapist appointments between randomisation and six-month follow-up), and the higher average cost of education services in the OST arm was driven by two high cost cases (one had 10 education welfare officer visits and another 11 school nurse visits between baseline and six-month follow-up). These high cost cases were kept in the analysis, as this was real world information and entirely plausible scenarios. Due to the presence of these high-cost cases, the cost differences need to be interpreted with caution.

**Table 2:** Average costs of service use between baseline and six-month follow-up by trial arm

	**Base case**		**Complete case**	
	**OST (*n* = 133), £ (95% CI)**	**CBT (*n* = 134), £ (95% CI)**	**OST (*n* = 94), £ (95% CI)**	**CBT (*n* = 96), £ (95% cI)**
**NHS and PSS**	**511.41 (348.69, 674.13)**	**544.00 (373.56, 714.43)**	**513.49 (351.10, 675.89)**	**549.05 (362.41, 735.70)**
Community-based services				
CAMHS related	206.53 (133.49, 279.57)	280.43 (211.09, 349.77)	210.47 (138.33, 282.61)	277.34 (207.50, 347.19)
Non-CAMHS related	47.61 (30.83, 64.39)	43.65 (25.66, 61.65)	48.15 (30.36, 65.94)	44.83 (25.60, 64.06)
Hospital-based services	168.37 (36.05, 300.69)	129.57 (16,71, 242.41)	163.12 (35.05, 291.18)	135.77 (14.04, 257.51)
Medications				
Mental health related	26.29 (12.60, 39.97)	23.99 (3.61, 44.36)	24.80 (10.66, 38.94)	23.45 (2.38, 44.52)
Non-mental health related	74.65 (31.79, 117.51)	68.12 (7.58, 128.67)	66.95 (19.89, 114.01)	67.66 (-4.58, 139.90)
**Education system related**	**25.87 (5.28, 46.46)**	**18.55 (1.64, 35.47)**	**28.77 (8.13, 49.42)**	**16.24 (2.97, 29.51)**
**Private expenses**	**26.18 (2.51, 49.85)**	**29.88 (12.96, 46.80)**	**24.30 (2.87, 45.73)**	**29.38 (11.90, 46.85)**
**Productivity**	**115.23 (73.65, 156.81)**	**136.13 (92.03, 180.23)**	**167.30 (123.24, 211.37)**	**213.56 (163.65, 263.47)**
**Total costs**	**678.69 (505.40, 851.98)**	**728.56 (549.15, 907.98)**	**733.88 (556.34, 911.41)**	**808.23 (610.74, 1,005.72)**

^Note : ^Education system related cost: we assume each appointment lasts 1 h based on the expert opinions

### Outcome measurements

Table 3 shows the mean EQ-5D-Y and CHU-9D utility scores across the two trial arms at each time point when missing scores were not imputed (complete case) and when missing scores were imputed (base case). As shown, in both arms, there was a small increase (0.03 to 0.04) in EQ-5D-Y utility scores from baseline to six-month follow-up point. Similar changes were also found in utility scores measured by the CHU-9D. Such small increases were observed in both the base and the complete case. After calculation using the area under the curve approach, it was found that OST produced similar mean QALYs (0.43 QALYs) to CBT, also the QALY estimates measured by EQ-5D-Y and CHU-9D were consistent. Further details for the responses of EQ-5D-Y and CHU-9D in each domain can be found in Additional file 1: Appendix 5 and Additional file 1: Appendix 6, respectively.

**Table 3:** Average EQ-5D-Y and CHU-9D utility scores by trial arm

Time point	Base case		Complete case	
	**OST (*n* = 133), mean (95% CI)**	**CBT (*n* = 134)** **mean (95% CI)**	**OST (*n* = 97), mean (95% CI)**	**CBT (*n* = 96)** **mean (95% CI)**
EQ-5D-Y				
Baseline	0.84 (0.82, 0.86)	0.85 (0.83, 0.87)	0.85 (0.83, 0.88)	0.85 (0.83, 0.87)
6 months	0.88 (0.86, 0.91)	0.88 (0.86, 0.90)	0.88 (0.86, 0.91)	0.88 (0.85, 0.90)
Total QALYs	0.43 (0.42, 0.44)	0.43 (0.42, 0.44)	0.43 (0.42, 0.44)	0.43 (0.42, 0.44)
CHU-9D				
Baseline	0.85 (0.83, 0.87)	0.86 (0.84, 0.87)	0.85 (0.83, 0.87)	0.86 (0.84, 0.88)
6 months	0.87 (0.85, 0.89)	0.89 (0.87, 0.91)	0.87 (0.85, 0.89)	0.89 (0.87, 0.91)
Total QALYs	0.43 (0.42, 0.44)	0.43 (0.43, 0.44)	0.43 (0.42, 0.44)	0.44 (0.43, 0.45)

### Primary economic analysis

**Fig. 1:**
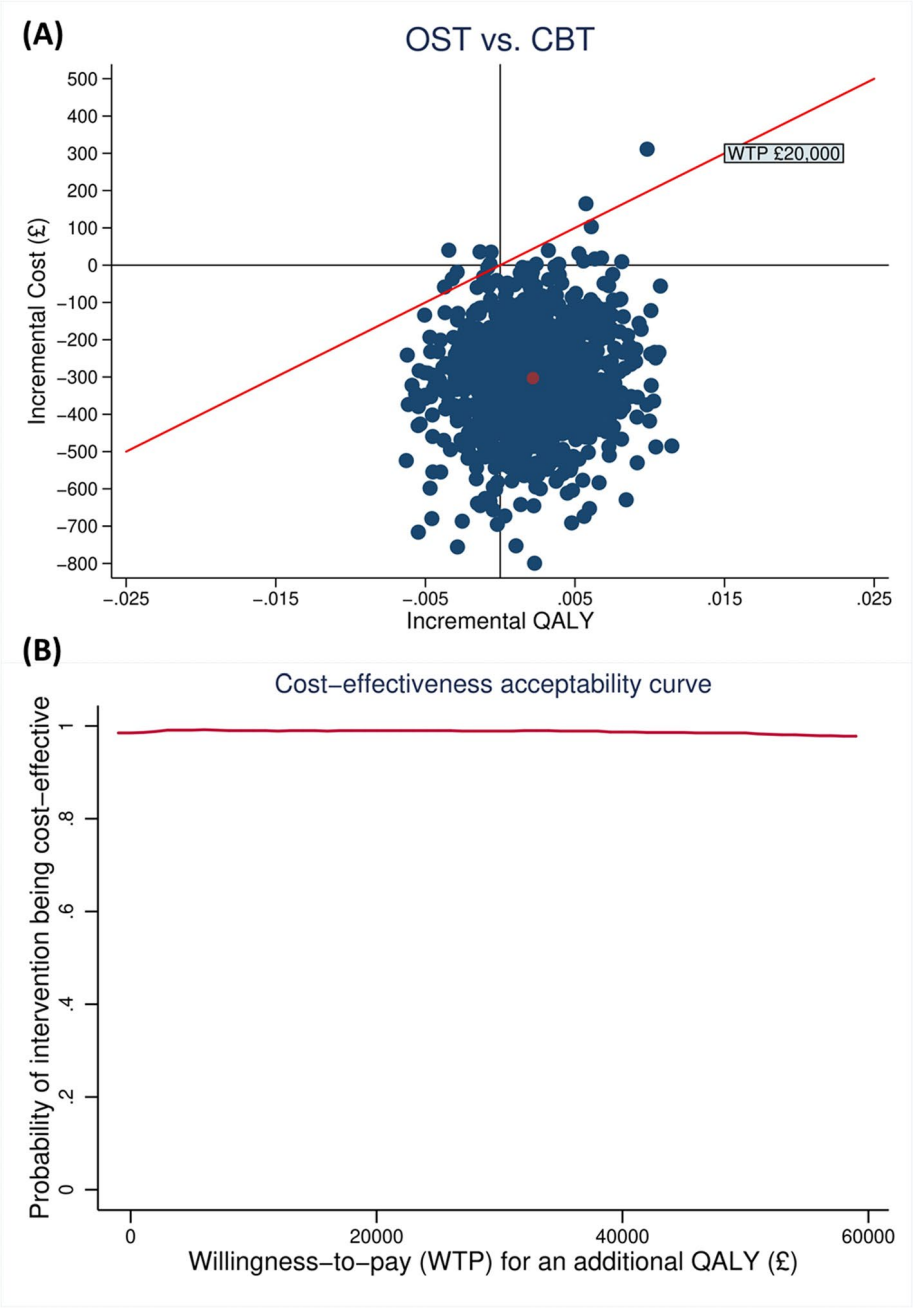
Cost-effectiveness plane and CEAC of primary analysis (outcome measures: QALY measured by EQ-5D-Y, costs from a NHS/PSS perspective)

The outcome of primary analysis was the incremental cost-effectiveness ratio (ICER) based on the base case. After accounting for the uncertainty and adjusting for the imbalanced utility scores and healthcare costs at baseline, on average, CYP with specific phobias receiving OST incurred £302.96 (95% CI £28.61 to £598.86) less costs and gained 0.002 (95% CI -0.004 to 0.008) QALYs than those receiving CBT, which is equivalent to an extra 0.73 days of perfect health. The 5,000 bootstrapped pairs of incremental costs and incremental QALYs from the regression were plotted on the CE plane (Fig. 1A). As shown in Fig. 1A, the bootstrapped estimates were largely below the £20,000 threshold line and sat in the third and fourth quadrant, indicating that OST was highly likely to be cost-saving. This finding was also confirmed by the CEAC (Fig. 1B). As shown in Fig. 1B, the estimated probability of OST being cost-effective is 0.98 when decision makers are willing to pay £20,000 for one QALY gained.

### Sensitivity analyses

**Fig. 2:**
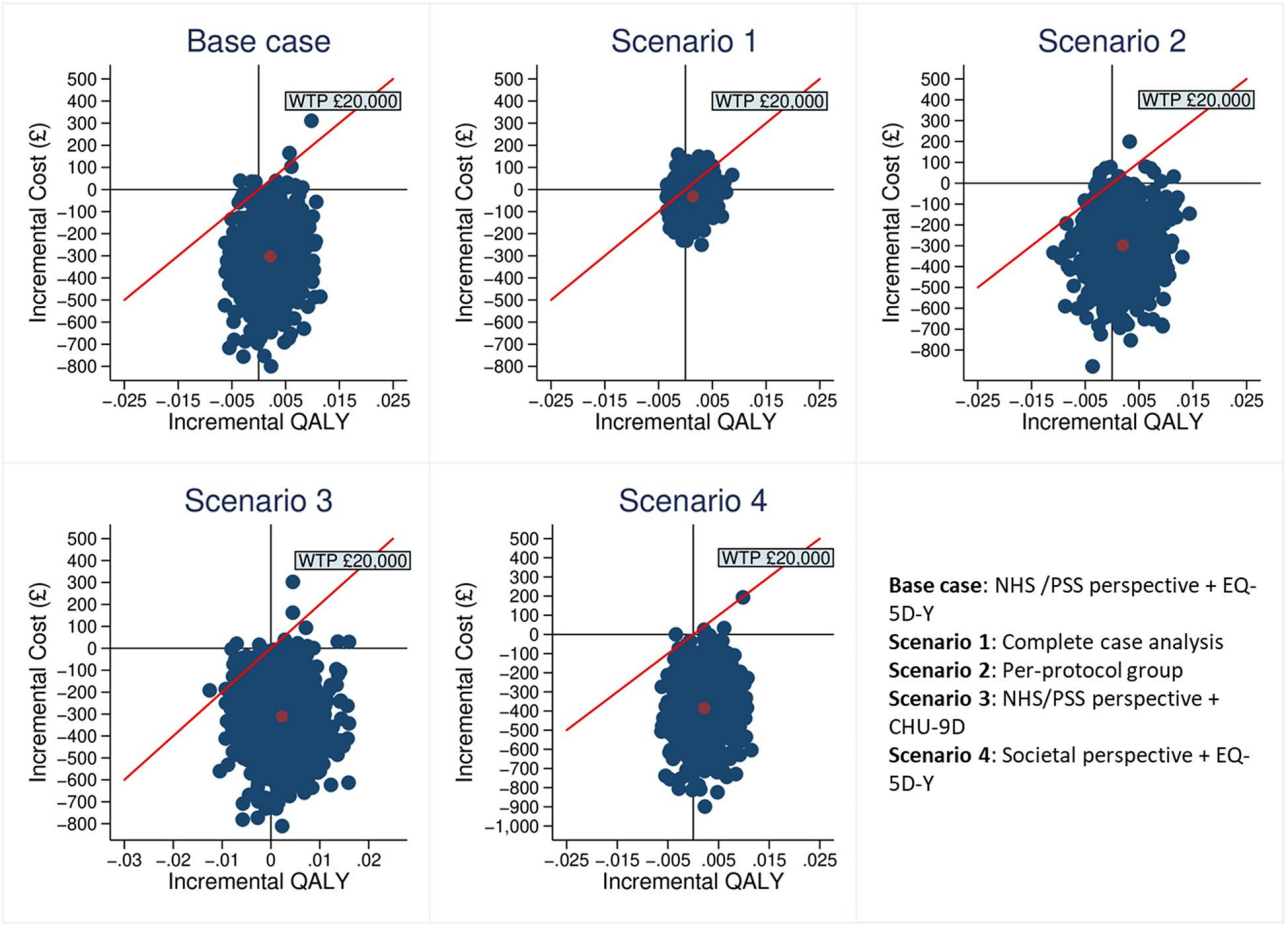
Cost-effectiveness planes of sensitivity analyses

To account for uncertainty in the cost and QALY estimations, a set of sensitivity analyses were conducted (Additional file 1: Appendix 7). The mean incremental cost and QALY estimates from the complete case were in keeping with the based-case scenario, yielding a negative cost per QALY gained. The same was observed among the CYP who received the intervention (for at least one session) within the trial follow-up period (*n* = 199, 98 from CBT and 101 from OST). Those who did not receive any intervention sessions (*n* = 67, 25%) due to the national lockdown caused by the COVID-19 pandemic in 2020 were excluded from the analysis. Other sensitivity analyses using a different cost perspective (societal perspective) and a different instrument (CHU-9D) to measure QALYs were also conducted. In the above mentioned sensitivity analyses, the mean ICER pairs lay below the recommended NICE threshold (£20,000–30,000/QALY gained), and the majority of bootstrapped estimates sat in the fourth quadrant and below the NICE threshold line (Fig. 2), confirming that OST was likely to be cost-saving compared to CBT.

## Discussion

### Principal findings

To our knowledge, this is the first study evaluating the cost-effectiveness of OST for CYP with specific phobias. Compared to multi-session CBT, OST slightly decreased the mean service use costs and increased the mean QALYs. This is evident in both primary and sensitivity analyses, which considered costs derived from various perspectives and QALYs measured by different instruments.

### Implications of study

Our study shows a small reduction in average total costs for OST. Compared to baseline, it was observed that both OST and CBT decreased resource use over time, but the cost difference (from either NHS/PSS or societal perspectives) between the two intervention groups at six-month follow-up was small. The cost difference was mainly driven by higher intervention costs, followed by higher CAMHS costs in the CBT group. The higher CAMHS costs were likely to have been caused by one high value (see Sect. 3.2: Costs), whereas the higher average intervention cost of CBT was caused by low unit cost but high frequency sessions involved. Both clinical scenarios were plausible in the real world given small numbers of CYP with specific phobia frequently needing high levels of therapeutic support for periods of time. Such cost difference or cost saving of OST within the six-month timeframe is expected to have been larger if CYP in the CBT arm did not pause or delay their sessions due to the COVID-19 pandemic. Additionally, it is possible that the cost saving of OST could be further increased if the intervention services were provided to a larger scale or implemented widely, as this can lead to lower average training cost (currently accounting for 33.8% of intervention costs, Additional file 1: Appendix 3).

Our study also found that CYP in the OST arm had maintained similar improvement in QALYs. Although both OST and CBT showed improvements in utility over time, the difference in QALYs between the two arms was small. After accounting for uncertainty and controlling for baseline characteristics, the QALY difference remained minimal (0.002 QALYs), indicating the non-inferiority property of OST in HRQoL compared to CBT. This is an encouraging finding, as OST requires one treatment session while multi-session CBT needs six to 12 weeks treatment. The improvement of HRQoL is considered to be larger if the impact of the convenience of OST can be captured. Finally, there is a concern regarding the 11 CYP under the age limit of eight who have completed the EQ-5D-Y self-complete version. Such implementation might create biases in the QALY estimates, although the number of CYP is small. It is encouraging that, based on the sensitivity analysis, the findings seem to have remained unaffected, regardless of the choice of instrument. The consistent estimates from EQ-5D-Y and CHU-9D not only ensured the robustness of our study results but also demonstrated high agreement between the two instruments when measuring HRQoL for CYP with specific phobias.

The results of the health economics analysis demonstrated that OST was highly likely to save costs and maintain similar improvement of QALYs compared to CBT, and the probability of OST being cost-effective was over 95% across all thresholds. On average, OST could potentially save around £300 per person from the NHS/PSS perspective. The saving is relatively small but may be relevant for commissioners when considering resource utilisation and specifically if multiplied by the number of CYP with specific phobias in the UK context. Owing to the fact that there are currently around 93,000 CYP with specific phobia in the UK (2,3) and around 50%—60% of specific phobias are treated with CBT [53, 54], the potential cost saving to the NHS could reach £14–17 million for the CYP population as a whole. One caveat to this is, for a variety of reasons, that only a proportion of CYP with specific phobia may be currently accessing treatments in the UK [55]. This adds further support to considering OST as an alternative treatment for CYP with specific phobias alongside the primary analysis of ASPECT which demonstrated OST to be non-inferior to CBT in terms of clinical effectiveness [18].

### Strengths and weakness

This is the first study evaluating the cost-effectiveness of OST for CYP with specific phobias. It is also the first study to compare the cost-effectiveness of OST with that of routinely delivered multi-session CBT. The importance of the study lies in the cost-effectiveness of OST being assessed by an evidence-based evaluation, rather than by simple assumptions based upon its shorter treatment period. The study results are likely to be useful to health policy makers, healthcare providers, CYP with specific phobias and their parent/guardians. Additionally, our study accounts for the costs measured from a range of perspectives and the QALYs measured by different instruments. The sensitivity analyses also explored the impact of missing data and the interruption of COVID-19 by analysing the complete case and the CYP who received at least one intervention sessions, respectively. The approach not only ensures the robustness of our findings but can help policy makers from different sectors to make informed decisions. Furthermore, unlike some studies that excluded certain types of specific phobia such as blood-injection-injury phobias [15], the present study investigated the full range of specific phobia types thereby representing the typical clinical population in CYP mental health services. This pragmatic study design allows the cost-effectiveness results to be applicable to real world clinical settings and represent specific phobias in CYP as a whole.

There were several limitations of the economic evaluation worthy of discussion. First, and most importantly, cases that could not receive allocated interventions or where interventions were interrupted due to the COVID-19 pandemic were a concern. The COVID-19 pandemic began in early 2020 and led to school closures, reduced service availability and difficulties in terms of in-person service delivery. The recruitment to ASPECT remained unaffected, but therapy delivery for some CYP was significantly impacted. In our study, 67 out of 267 CYP did not receive any intervention during the study period. This is partially because COVID-19 stopped many clinical services offering routine face to face treatments and partially because of CYP withdrawal and issues with services (i.e. staff absence and delays in starting treatment). Although this could potentially introduce bias to our results, the sensitivity analyses (see Sect. 3.5) on those who had interventions within the six-month follow-up period showed that our findings were largely similar to our primary analysis, thus supporting the robustness of the results. Second, service use data were collected retrospectively, and there may have been recall accuracy problems. However, this is unlikely to have affected one group more than another. Hence, the study comparison results are likely to remain unchanged. Finally, our cost results can be influenced by the “did not attend” appointments (DNAs), as a DNA of OST has a greater financial impact compared to a DNA of CBT (OST typically lasts around three hours; while CBT is typically an hour long). Although the costs of DNAs can play a role in our study and can be considerable to the NHS [56], our study did not take these costs into account due to data constraints. This decision aligns with the NHS England’s 2020 National Cost Collection guidance for mental health, which advises that missed appointments/ DNAs should not be included in the cost collection [57].

### Future research

Our study measured the short-term cost-effectiveness of OST for CYP with specific phobia between baseline and six-months follow-up. Although the long-term cost-effectiveness of OST was outside the scope of the current study, a model-based economic evaluation study would be desirable in future research to allow life-time cost-effectiveness and CYP’s lost productivity during adulthood to be measured.

## Conclusion

Both our primary and sensitivity analyses suggest that OST is likely to save cost and maintain similar QALYs compared to multi-session CBT for CYP with specific phobias. The findings will be of interest to policy makers, commissioners, NHS health and social care providers, local authorities, and families with an interest in child and adolescent mental health.

## Supplementary Information

**Additional file 1.**

## Data Availability

The data used for the current study may be made available by the corresponding author upon reasonable request with justification.

## Abbreviations

ADIS: Anxiety disorder interview schedule
ASPECT: Alleviating specific phobias experienced by children trial
BAT: Behavioural avoidance task
CAIS: Child Anxiety Impact Scale
CAMHS: Child and adolescent mental health services
CBT: Cognitive behavioural therapy
CE: Cost-effectiveness
CEAC: Cost-effectiveness acceptability curve
CHU-9D: Child Health Utility-9D
COMIC-RU: Child oriented mental health interventions centre – resource use
CRS: Clinician severity rating
CUA: Cost utility analysis
CYP: Children and young people
DNA: Did not attend EQ-5D-Y: youth version of the EQ-5D
HRQoL: Health related quality of life
ICER: Incremental cost-effectiveness ratio
NHS/PSS: NHS and personal social service
OST: One session treatment
PSS: Personal social services
PSSRU: Personal social services research unit
QALY: Quality adjusted life years
RCADS: Revised children’s anxiety and depression scale
RCT: Randomised controlled trial
SURE: Seemingly unrelated regression equations
WTP: Willingness-to-pay
