# Supplementary material for: Cost and effectiveness of one session treatment (OST) for children and young people with specific phobias compared to multi-session cognitive behavioural therapy (CBT): results from a randomised controlled trial
Source: BMC Psychiatry. 2022 Aug 12;22:547. doi: 10.1186/s12888-022-04192-8 (PMC9372970; doi:10.1186/s12888-022-04192-8)
Supplement: Supplementary file 1 — Additional file 1. [file 12888_2022_4192_MOESM1_ESM.pdf]

Appendix 1 Participant flow

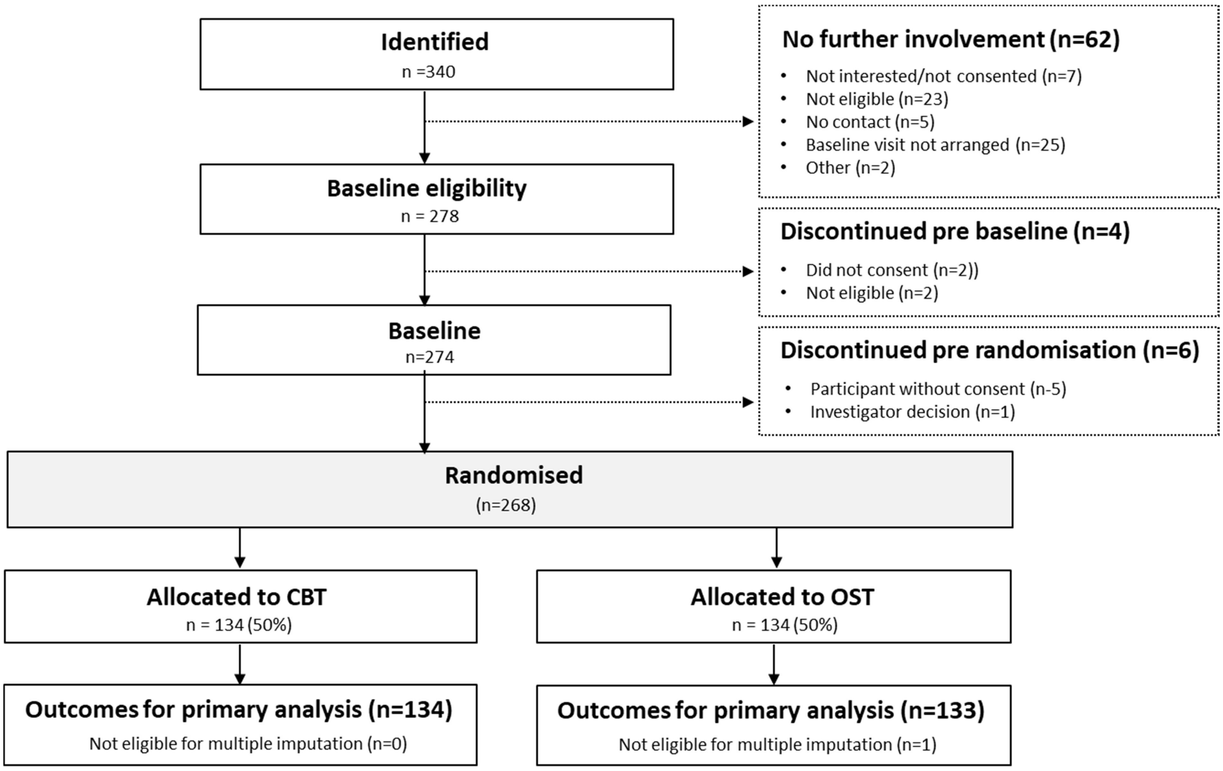

Appendix 2 missing pattern

	Baseline			6-month follow-up			Baseline + 6-month follow-up		
	Total (n=267) n (%)	OST (n=133) n (%)	CBT (n=134) n (%)	Total (n=267) n (%)	OST (n=133) n (%)	CBT (n=134) n (%)	Total (n=267) n (%)	OST (n=133) n (%)	CBT (n=134) n (%)
**EQ-5D-Y and resource use questionnaire**									
Completed both	266 (99.6)	132 (99.2)	134 (100.0)	191 (71.5)	95 (71.4)	96 (71.6)	190 (71.2)	94 (70.7)	96 (71.6)
Resource use only	1 (0.4)	1 (0.8)	-	2 (0.7)	1 (0.8)	1 (0.8)			
EQ-5D-Y only	-	-	-	2 (0.7)	2 (1.5)	-			
Completed none of both	-	-	-	72 (27.0)	35 (26.3)	37 (27.6)			
**EQ-5D-Y**									
No missing	267 (100.0)	133 (100.0)	134 (100.0)	193 (72.3)	97 (72.9)	96 (71.6)	193 (72.3)	97 (72.9)	96 (71.6)
**Resource use questionnaire**									
No missing	266 (99.6)	132 (99.2)	134 (100.0)	193 (72.3)	96 (72.2)	97 (72.4)	192 (71.9)	95 (71.4)	97 (72.4)

Appendix 3 Intervention costs by trial arm

	OST		CBT	
	**Total cost (£)**	**Cost per session per CYP (£)**	**Total cost (£)**	**Cost per session per CYP (£)**
Training costs				
Train the trainers				
Venue	£140.00	£1.47	-	-
Refreshment	£335.00	£1.45	-	-
Consumable costs	£0.00	£0.00	-	-
Trainer's travel costs	£6,117.00	£26.48	-	-
Trainer fee	£1,940.00	£8.40	-	-
Total	£8,392.00	£36.33	-	-
Therapist training				
Venue	£160.00	£0.69	-	-
Refreshment costs	£732.40	£3.17	-	-
Consumable costs	£264.00	£1.14	-	-
Trainer's travel costs	£317.20	£1.37	-	-
Trainer fee	£4,500.00	£19.48	-	-
Total	£5,973.60	£25.86	-	-
Intervention delivery costs				
Intervention (staff time)				
Therapy session	£19,054.33	£82.49	£29,908.85	£45.45
Administration / preparation	£5,151.17	£22.30	£5,507.37	£8.40
Additional resource				
Second therapist	£2,037.00	£8.82	£550.67	£0.84
Assistant / nurse	£331.88	£1.44	£55.13	£0.09
Stimuli	£503.84	£2.18	£811.36	£1.23
Supervision*	£1,119.77	£4.84	£1,717.01	£2.61
Total	£28,197.98	£122.07	£38,553.38	£58.59

*The calculation method and unit cost were based on Wiles et al. study: Wiles N, Thomas L, Abel A et al. Clinical effectiveness and cost-effectiveness of cognitive behavioural therapy as an adjunct to pharmacotherapy for treatment-resistant depression in primary care: the CoBalT randomised controlled trial. Health Technol Assess. 2014 May;18(31):1-167.

Appendix 4 Average service use by trial arm (complete case, n=139)

	Unit	Baseline		6 months	
		OST, N=94<br>Mean<br>(min-max)<br>(95% CI)	CBT, N=96<br>Mean<br>(min-max)<br>(95% CI)	OST, N=94<br>Mean<br>(min-max)<br>(95% CI)	CBT, N=96<br>Mean<br>(min-max)<br>(95% CI)
NHS and PSS					
Community-based services					
CAMHS related					
Child psychiatrist	Appointment	0.17 (0-2)<br>(0.07 – 0.27)	0.12 (0-3)<br>(0.22 – 0.21)	0.15 (0-3)<br>(0.04 – 0.26)	0.18 (0-5)<br>(0.16 – 0.34)
Child psychotherapist	Appointment	0.24 (0-8)<br>(0.01 – 0.47)	0.20 (0-18)<br>(-0.18 – 0.56)	0.07 (0-3)<br>(-0.01 – 0.16)	0.34 (0-25)<br>(-0.04 – 0.23)
Child psychologist	Appointment	0.06 (0-3)<br>(-0.01 – 0.14)	0.35 (0-11)<br>(0.07 – 0.64)	0.30 (0-8)<br>(0.07 – 0.52)	0.30 (0-8)<br>(0.04 – 0.57)
Mental health nurse	Appointment	1.91 (0-24)<br>(1.17 – 0.65)	1.03 (0-9)<br>(0.65 – 1.41)	1.97 (0-27)<br>(0.97 – 2.99)	3.09 (0-24)<br>(2.12 – 4.07)
Family therapist	Appointment	-	0.01 (0-1)<br>(-0.01 – 0.03)	0.02 (0-2)<br>(-0.02 – 0.06)	-
Non-CAMHS related					
GP	Appointment	1.65 (0-20)<br>(1.12 – 2.17)	1.17 (0-10)<br>(0.86 – 1.48)	0.55 (0-10)<br>(0.29 – 0.81)	0.73 (0-8)<br>(0.43 – 1.04)
Nurse	Appointment	0.40 (0-11)<br>(0.14 – 0.67)	0.44 (0-12)<br>(0.13 – 0.74)	0.17 (0-3)<br>(0.06 – 0.28)	0.39 (0-12)<br>(0.05 – 0.72)
Community paediatrician	Appointment	0.18 (0-4)<br>(0.05 – 0.32)	0.08 (0-2)<br>(0.01 – 0.15)	0.04 (0-2)<br>(-0.01 – 0.09)	0.03 (0-2)<br>(-0.01 – 0.08)
Child development centre	Visit	0.17 (0-12)<br>(-0.09 – 0.42)	0.01 (0-1)<br>(-0.01 – 0.03)	0.08 (0-3)<br>(-0.00 – 0.17)	0.01 (0-1)<br>(-0.01 – 0.03)
Helpline / NHS-direct	Call	0.05 (0-2)<br>(-0.00 – 0.12)	0.05 (0-1)<br>(0.01 – 0.10)	0.06 (0-3)<br>(-0.01 – 0.14)	0.04 (0-2)<br>(-0.01 – 0.09)
Walk-in centre	Visit	0.13 (0-2)<br>(0.04 – 0.22)	0.11 (0-2)<br>(0.04 – 0.19)	0.10 (0-3)<br>(0.01 – 0.18)	0.10 (0-3)<br>(0.02 – 0.18)
Social worker	Visit	0.20 (0-8)<br>(0.00 – 0.40)	0.25 (0-7)<br>(0.05 – 0.45)	0.07 (0-3)<br>(-0.02 – 0.17)	0.04 (0-4)<br>(-0.04 – 0.12)
Hospital-based services					
Emergency services	Visit	0.17 (0-2)<br>(0.08 – 0.26)	0.09 (0-2)<br>(0.03 – 0.16)	0.12 (0-2)<br>(0.04 – 0.20)	0.07 (0-2)<br>(0.01 – 0.14)
Inpatient admission	Admission	0.04 (0-2)<br>(-0.01 – 0.09)	0.03 (0-2)<br>(-0.01 – 0.08)	0.02 (0-1)<br>(-0.01 – 0.05)	0.04 (0-2)<br>(-0.02 – 0.10)
Outpatient visit	Attendance	0.24 (0-5)	0.26 (0-8)	0.23 (0-3)	0.28 (0-4)

		(0.07 – 0.42)	(0.07 – 0.45)	(0.11 -0.36)	(0.13 – 0.44)
Medication					
Mental health related	Type	0.22 (0-3)	0.18 (0-4)	0.22 (0-3)	0.18 (0-4)
		(0.11 – 0.34)	(0.05 – 0.31)	(0.11 – 0.34)	(0.05 – 0.31)
Non-mental health related	Type	0.48 (0-7)	0.38 (0-8)	0.48 (0-7)	0.38 (0-8)
		(0.25 – 0.71)	(0.17 – 0.58)	(0.25 – 0.71)	(0.17 – 0.58)
Education system related					
Education psychologist	Appointment	0.13 (0-7)	0.04 (0-1)	0.07 (0-7)	0.07 (0-5)
		(-0.03 – 0.28)	(0.00 – 0.08)	(-0.05 – 0.20)	(-0.03 – 0.18)
Education welfare officer	Appointment	0.06 (0-3)	0.32 (0-20)	0.17 (0-10)	0.05 (0-2)
		(-0.01 – 0.14)	(-0.11 – 0.76)	(-0.02 – 0.36)	(-0.01 – 0.11)
School nurse	Appointment	0.20 (0-5)	0.67 (0-20)	0.13 (0-11)	0.01 (0-1)
		(0.03 – 0.38)	(0.08 – 1.26)	(-0.00 – 0.26)	(-0.01 – 0.03)
School counsellor	Appointment	1.84 (0-30)	0.60 (0-10)	1.04 (0-25)	0.60 (0-20)
		(0.80 – 2.88)	(0.20 – 1.00)	(0.20 – 1.88)	(0.04 – 1.17)
Private expense					
Self-paid treatment	Session	0.44 (0-24)	0.19 (0-10)	0.04 (0-2)	0.02 (0-2)
		(-0.10 – 0.97)	(-0.08 – 0.45)	(-0.02 – 0.10)	(-0.02 – 0.06)
Travel for intervention	Hour	-	-	1.85 (0-7)	5.31 (0-16)
				(1.64 – 2.06)	(4.57 – 6.04)
Productivity					
Parental productivity	Day	2.73 (0-63)	1.15 (0-19)	0.89 (0-10)	0.82 (0-12)
		(1.10 – 4.36)	(0.56 – 1.74)	(0.48 – 1.30)	(0.43 – 1.22)
Productivity for intervention	Hour	-	-	5.32 (0-16	11.01 (0-33)
				(4.79 – 5.85)	(9.55 – 12.47)

Appendix 5 EQ-5D-Y responses by trial arms by data collection time points

	Baseline			6 months		
	Level 1	Level 2	Level 3	Level 1	Level 2	Level 3
**OST (n=97)**	n (%)	n (%)	n (%)	n (%)	n (%)	n (%)
Mobility	87 (89.7)	9 (9.3)	1 (1.0)	89 (91.8)	7 (7.2)	1 (1.0)
Self-care	86 (88.7)	7 (7.2)	4 (4.1)	87 (89.7)	10 (10.3)	-
Usual activity	63 (64.9)	31 (32.0)	3 (3.1)	79 (81.4)	16 (16.5)	2 (2.1)
Pain/discomfort	61 (62.9)	36 (37.1)	-	70 (72.2)	24 (24.7)	3 (3.1)
Anxiety/depression	44 (45.4)	43 (44.3)	10 (10.3)	55 (56.7)	36 (37.1)	6 (6.2)
	**Baseline**			**6 months**		
	**Level 1**	**Level 2**	**Level 3**	**Level 1**	**Level 2**	**Level 3**
**CBT (n=96)**	**n (%)**	**n (%)**	**n (%)**	**n (%)**	**n (%)**	**n (%)**
Mobility	82 (85.5)	13 (13.5)	1 (1.0)	86 (89.6)	10 (10.4)	-
Self-care	85 (88.5)	11 (11.5)	-	86 (89.6)	10 (10.4)	-
Usual activity	65 (67.7)	27 (28.1)	4 (4.2)	76 (79.2)	19 (19.8)	1 (1.0)
Pain/discomfort	61 (63.5)	30 (31.3)	5 (5.2)	69 (71.9)	25 (26.0)	2 (2.1)
Anxiety/depression	43 (44.8)	47 (49.0)	6 (6.2)	49 (51.0)	40 (41.7)	7 (7.3)

Note: Level 1: none, Level 2: some, Level 3: extreme

Appendix 6 CHU-9D responses by trial arms by data collection time points

	Baseline					6 months				
**OST (n=45)**	**Level 1**	**Level 2**	**Level 3**	**Level 4**	**Level 5**	**Level 1**	**Level 2**	**Level 3**	**Level 4**	**Level 5**
Worried	20 (44.4)	16 (35.6)	7 (15.6)	-	2 (4.4)	22 (48.9)	12 (26.7)	5 (11.1)	3 (6.7)	3 (6.7)
Sad	32 (71.1)	8 (17.8)	2 (4.4)	2 (4.4)	1 (2.2)	29 (64.4)	6 (13.3)	5 (11.1)	4 (8.9)	1 (2.2)
Annoyed	27 (60.0)	13 (28.9)	4 (8.9)	-	1 (2.2)	27 (60.0)	10 (22.2)	5 (11.1)	-	3 (6.7)
Tired	9 (20.0)	15 (33.3)	8 (17.8)	5 (11.1)	8 (17.8)	12 (26.7)	13 (28.9)	9 (20.0)	5 (11.1)	6 (13.3)
Pain	28 (62.2)	6 (13.3)	6 (13.3)	3 (6.7)	2 (4.4)	21 (46.7)	11 (24.4)	4 (8.9)	2 (4.4)	7 (15.6)
Sleep	21 (46.7)	12 (26.7)	5 (11.1)	3 (6.7)	4 (8.9)	20 (44.4)	3 (6.7)	7 (15.6)	9 (20.0)	6 (13.3)
Daily routine	23 (51.1)	12 (26.7)	4 (8.9)	3 (6.7)	3 (6.7)	19 (42.2)	11 (24.4)	7 (15.6)	4 (8.9)	4 (8.9)
Work	27 (60.0)	15 (33.3)	2 (4.4)	1 (2.2)	-	23 (51.1)	11 (24.4)	7 (15.6)	1 (2.2)	3 (6.7)
Able to join activities	23 (51.1)	6 (13.3)	10 (22.2)	3 (6.7)	3 (6.7)	10 (22.2)	14 (31.1)	10 (22.2)	9 (20.0)	2 (4.4)

	Baseline					6 months				
**CBT (n=51)**	**Level 1**	**Level 2**	**Level 3**	**Level 4**	**Level 5**	**Level 1**	**Level 2**	**Level 3**	**Level 4**	**Level 5**
Worried	34 (66.7)	8 (15.7)	3 (5.9)	2 (3.9)	4 (7.8)	33 (64.7)	6 (11.8)	7 (13.7)	1 (2.0)	4 (7.8)
Sad	40 (78.4)	5 (9.8)	2 (3.9)	1 (2.0)	3 (5.9)	37 (72.5)	5 (9.8)	5 (9.8)	1 (2.0)	3 (5.9)
Annoyed	37 (72.5)	7 (13.7)	4 (7.8)	2 (3.9)	1 (2.0)	34 (66.7)	10 (19.6)	5 (9.8)	-	2 (3.9)
Tired	14 (27.5)	19 (37.3)	5 (9.8)	3 (5.9)	10 (19.6)	17 (33.3)	19 (37.3)	4 (7.8)	7 (13.7)	4 (7.8)
Pain	33 (64.7)	9 (17.6)	3 (5.9)	1 (2.0)	5 (9.8)	28 (54.9)	9 (17.6)	7 (13.7)	3 (5.9)	4 (7.8)
Sleep	26 (51.0)	10 (19.6)	6 (11.8)	3 (5.9)	6 (11.8)	23 (45.1)	8 (15.7)	8 (15.7)	4 (7.8)	8 (15.7)
Daily routine	29 (56.9)	8 (15.7)	4 (7.8)	4 (7.8)	6 (11.8)	24 (47.1)	15 (29.4)	7 (13.7)	3 (5.9)	2 (3.9)
Work	34 (66.7)	7 (13.7)	6 (11.8)	1 (2.0)	3 (5.9)	33 (64.7)	10 (19.6)	5 (9.8)	3 (5.9)	-
Able to join activities	30 (58.8)	6 (11.8)	5 (9.8)	4 (7.8)	6 (11.8)	24 (47.1)	8 (15.7)	6 (11.8)	8 (15.7)	5 (9.8)

Note: Level 1: No, Level 2: A little bit, Level 3: A bit, Level 4: Quite a lot, Level 5 Very

Appendix 7 Sensitivity analyses

OST vs. CBT	Incremental costs (£), (95% CI)	Incremental QALYs (95% CI)	ICER (£/QALY gained), (95% CI)	Probability of OST being cost effective at WTP threshold of £20,000/QALYs
Base-case	-302.96<br>(-28.61, -598.86)	0.002<br>(-0.004, 0.008)	Dominant	98%
Scenario 1: Complete case analysis from NHS/PSS perspective	-31.10<br>(-154.21, 88.14)	0.001<br>(-0.002, 0.005)	Dominant	81%
Scenario 2: CUA on CYP who received at least one intervention session from NHS/PSS perspective	-298.40<br>(-599.68, -23.90)	0.002<br>(-0.005, 0.009)	Dominant	99%
Scenario 3: CUA (utility measured by CHU9D)	-310.64<br>(-605.07, -44.10)	0.002<br>(-0.007, 0.012)	Dominant	98%
Scenario 4: CUA (cost from societal perspective)	-384.81<br>(-685.01, -107.16)	0.002<br>(-0.004, 0.008)	Dominant	99%
